# Supplementary material for: Saccharomyces boulardii (CNCM I-745) improves intestinal damage in sepsis by remodeling intestinal flora structure
Source: Front Cell Infect Microbiol. 2025 Jul 31;15:1602792. doi: 10.3389/fcimb.2025.1602792 (PMC12350329; doi:10.3389/fcimb.2025.1602792)
Supplement: Supplementary file 1 [file DataSheet1.zip › summary/summary/7_Differential_analysis/3_Indicator_result/3_Class/Class_indicator_Group.pdf]

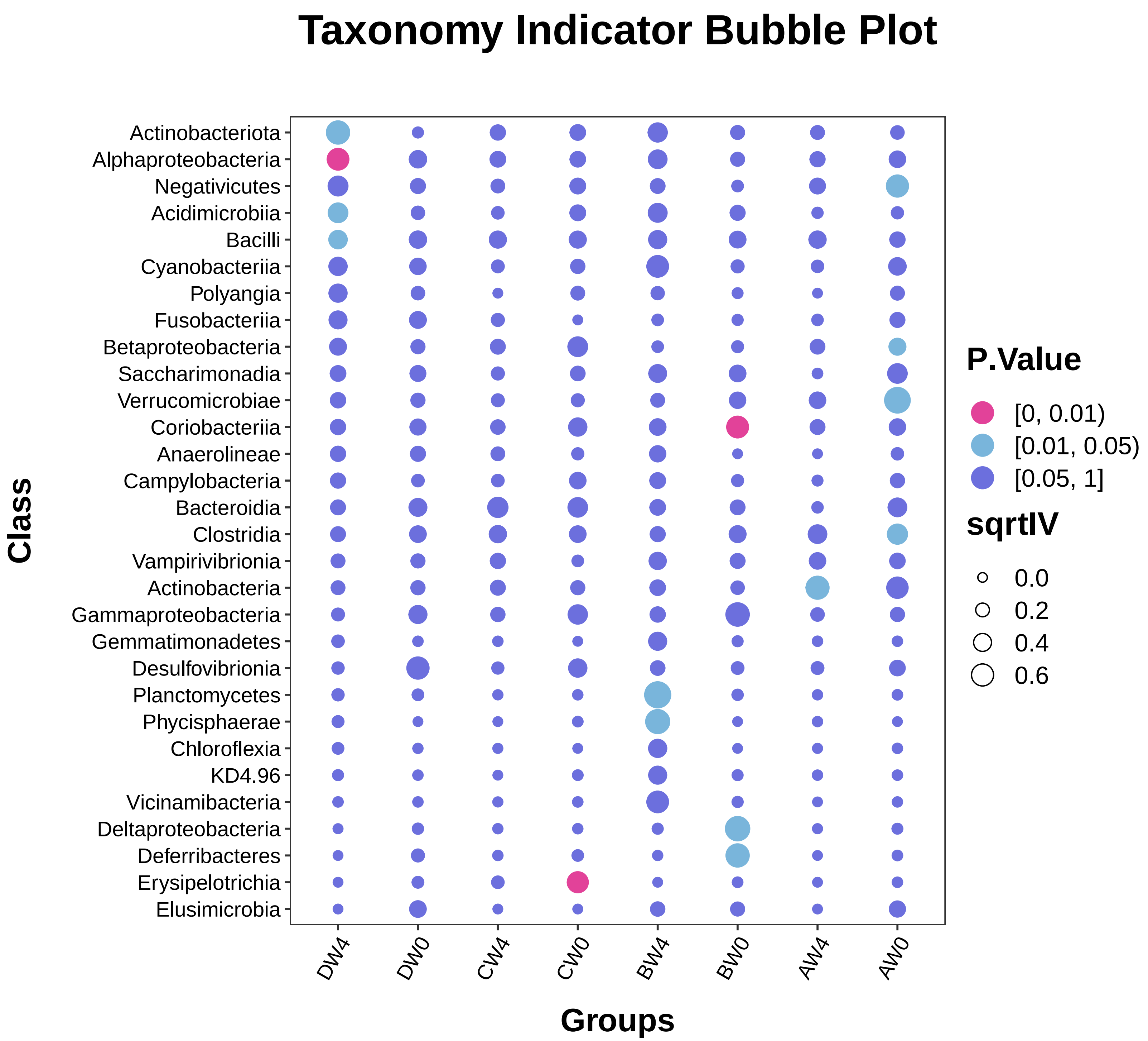
Taxonomy Indicator Bubble Plot
Class
Actinobacteriota
Alphaproteobacteria
Negativicutes
Acidimicrobiia
Bacilli
Cyanobacteriia
Polyangia
Fusobacteriia
Betaproteobacteria
Saccharimonadia
Verrucomicrobiae
Coriobacteriia
Anaerolineae
Campylobacteria
Bacteroidia
Clostridia
Vampirivibrionia
Actinobacteria
Gammaproteobacteria
Gemmatimonadetes
Desulfovibrionia
Planctomycetes
Phycisphaerae
Chloroflexia
KD4.96
Vicinamibacteria
Deltaproteobacteria
Deferribacteres
Erysipelotrichia
Elusimicrobia
DW4
DW0
CW4
CW0
BW4
BW0
AW4
AW0
Groups
P.Value
[0, 0.01)
[0.01, 0.05)
[0.05, 1]
sqrtIV
0.0
0.2
0.4
0.6
